# Hints for the Sick-Room

**Published:** 1888-09-15

**Authors:** M. M. Basil

**Affiliations:** Author of "The Commoner Accidents to Life and Limb."


### Hints for the Sick-room.

By M. M. Basil, M.A., M.B., C.M., Author of "The Commoner Accidents to Life and Limb."

ADMINISTRATION OF MEDICINES (*continued*).

Medicinal enemata are given either to act on diseased conditions of the bowel and neighbouring organs, or to act on the constitution by absorption from the bowel. If the rectum itself is diseased and irritable, the enema must be of very small bulk—not more than an ounce when this means of medication must be continued for some days. If the rectum itself is not irritable, the enema may consist of two to four ounces—not more. In certain conditions it is best to empty the bowel of flatus by introducing a simple indiarubber tube first, then to wash out the rectum with warm water and inject the medicinal enema half an hour after all the water has been expelled. At any rate, you must be satisfied that the bowel is not loaded with hard fæces before a medicinal injection is thrown in, as otherwise the action of the drug will be materially retarded if not entirely prevented.

For dietetic and medicinal enemata a glass syringe provided with a short rubber tube is preferable to any form of apparatus made of indiarubber; for injecting starchy preparation a glass syringe is a necessity.

The use of the bed-pan may be conveniently described in this connexion. In their selection, preference should be given to slipper-shaped or round forms. Metallic ones should be rejected, as they are apt to cause bed-sores, and cannot be kept free from smell. Bed-pans should have flannel covers, and in cold weather should be warmed immediately before use. They should be provided with lids, which may be metallic or earthenware, but not wood. They must be removed out of the sick-room at once. All the foul material which is usually deposited on the inner surface of the lid passes into the air of the sick-room if the lid is wanting. This point must be attended to for your own safety, if on no other grounds. As we shall see later on, some diseases are essentially fæcal diseases—that is, they spread from the affected individual to his neighbours by means of the intestinal evacuations. Naturally the person who runs the greatest risk of being poisoned by the infectious excretions is the nurse in constant attendance in the room. The stools in typhoid fever, and in all suspicious diseases where diarrhœa is a symptom, must be carefully disinfected. We shall examine this question in detail when the nursing of fevers comes under consideration.

Before introducing the bed-pan, be certain that it is perfectly sound; serious accidents may result if it should be smashed when under the patient. In cases where the individual is heavy, helpless, or prone to bed-sores, the edges of the pan should be lubricated, and it should be introduced between the back of the nurse's arm and the bed, while the patient's pelvis is lifted up with the hand. The edges of the bed-pan must not come in contact with the patient's body during the introduction. It should be well rinsed soon after use, and some disinfectant solution left in it.

In a ward screens must almost invariably be arranged during the use of the bed-pan. In the night its presence under the patient must not be forgotten. If the patient is helpless or stupid, and has to be raised through the night, attention must be paid to having her put into bed again, otherwise serious results from exposure to cold and other causes may supervene.

The introduction of the catheter in the female is an operation with which every nurse must make herself familiar. The procedure in itself is perfectly free from any danger, and hence no single case where it leads to serious results can be justified as an unavoidable accident risked with full knowledge of its possible supervention. It therefore behoves us to know all that is essential for the prevention of any painful results.

The introduction of the catheter is a part of nursing which can only be learned by practice; no description of the process can possibly teach it to anyone. Still, however, an intelligent knowledge of many of the steps of the operation will be of advantage in practice.

The most convenient catheter to use is an elastic male one (No. 8). An indiarubber tube of several inches may be slipped over its end to conduct the urine into a vessel placed under the bed. Metallic catheters had better be avoided, but where their employment becomes compulsory it is best to slip them through the fingers from point to end, to be sure that they are not likely to wound the urethra by roughness at any spot. Before introducing the elastic catheter, too, you must be sure that it is not cracked at any point, especially where the eye of it is situated. Should the point break off in the bladder, there ought to be no delay in informing the surgeon of the accident.

The catheter must be perfectly clean, not only outside, but particularly on the inner surface and the space between the eye and the blind extremity. The oil for lubricating it must not contain too large a proportion of carbolic acid: 1 in 40 is sufficient, provided cleanliness is systematically insisted upon; 1 in 20 is the strongest that ought to be used. Inflammation of the urethra, or bladder, may be the consequence of using oil containing a larger proportion of carbolic acid.

The position of the patient is a matter of secondary importance Sims' position is the best if it can be assumed, since the parts can be readily exposed in this way if such a step is thought advisable, but it is equally easy to pass the catheter while the patient is lying simply on the side or on the back, with the thighs flexed or even fully extended.

The index finger of the left hand acts as your guide in determining the position of the urethral orifice. The catheter is held very lightly between the thumb and index finger of the right hand. No force or firmness of touch is at all admissible here. Complete failure in passing the catheter is an issue far preferable to its successful accomplishment effected with force or undue perseverance.

The best guide to the position of the urethral opening is the touch of the educated finger. A soft velvety projection above the vaginal entrance, when it can be felt, is a good landmark. It is also important to remember that the urethra is just under the pubic symphysis, and can be felt here through the anterior vaginal wall. The introduction of the left index finger into the vagina and the apposition of its volar surface to the pubic symphesis will prevent the catheter from entering the vagina, and may help it to be guided into the urethra. These are the usual guides for introducing the instrument without exposing the parts. It is no doubt easy, after some practice, to introduce the instrument under the sheets. Whether in all cases, or in most cases, it is expedient or justifiable to do so is another question. Certainly the principle that should guide you in practice is to expose the parts, rather than to run any risk of doing harm by groping about blindly. Then, again, in a large number of cases, where the introduction of the catheter is necessary, it is advisable, as a preliminary step of the operation, to expose the parts, and thoroughly to clean and disinfect the area round the urethral orifice before the catheter is introduced. Such are nearly all cases where the operation is necessitated after the lying-in condition among the poor of large towns and in cases where there is profuse purulent discharge from the parts. These two groups form a large portion of cases that come under observation in nursing among the poor of cities.

(*To be continued.*)

